# Reduction of a Femoral Dislocation of Six Months’ Standing, by Manipulation

**Published:** 1857-03

**Authors:** Martial Dupierris

**Affiliations:** Havana, Cuba


ART. VI.—*Reduction of a Femoral Dislocation, of Six Months' Standing, by Manipulation.* By MARTIAL DUPIERRIS, M.D., of Havana, Cuba.

SIR ASTLEY COOPER, in his surgical work, asserts it to be imprudent to attempt the reduction of a shoulder dislocation after three months, and of the hip after eight weeks, except in patients of very lax fibre or advanced age. But this great master admitted the existence of exceptions, and reported a case of dislocation of the head of the femur upon the dorsum ilii, reduced after the lapse of five years. The following case, although not very extraordinary, is instructive, as forming an exception to the rule laid down by authors of the highest standing.

The ship "Sword Fish," arrived at this port, on the 4th of June last, with a cargo consisting of three hundred and seventy-five Chinese colonists. Among the sick on board was a boy, about sixteen years old, called A-sin, who was taken to the infirmary. His skin was hot and dry; his tongue was parched; his pulse was very frequent and sometimes intermittent; he had a diarrhœa; and he lay upon his back with his thighs flexed upon the abdomen at a right angle, and his legs upon his thighs. He complained much of pain in the abdomen, and of excessive thirst. Our interpreter not understanding the patient's language very well, we deferred any further examination, hoping to procure shortly some one who could obtain from him a reliable history of the case. In the meantime treatment was ordered with a view to subdue the febrile action, and arrest the gastro-intestinal disorder.

After a month's careful attention, the fever was broken, but returned at irregular intervals in the form of short paroxysms, characterized by heat and perspiration; and the diarrhœa and intestinal irritation had entirely disappeared. Upon now being requested to get out of bed, he replied that it was impossible; and when he was lifted up by two nurses, he cried out with pain. I now discovered that when he was held in the erect posture, only the right foot touched the floor, the left being raised to the distance of seven centimetres; that the toe of the latter and the corresponding knee were inverted; and that his cries resulted from pain, produced by the nurse in an effort to make the left foot rest upon the floor. The peculiar deformity of the limb led me at once to suspect the existence of a coxo-femoral dislocation, and, upon farther examination, my suspicions were confirmed by finding the head of the left femur upon the corresponding dorsum ilii.

Upon questioning the patient, through a new interpreter, in regard to the cause of the accident, I learned that before leaving China, while carrying two buckets of water suspended from the extremities of a pole placed across his shoulders, he slipped and fell upon his left side; that, being unable to rise, he was carried to the trading house, where he was treated by a native physican, and, after several days, was able to walk with a stick, without being able, however, to get his foot to the ground. After going on board the vessel, his sufferings increased, and he was obliged to keep upon his back during the entire voyage. In the meantime, he lost his appetite, and his bowels became deranged.

I have already described the condition of the patient upon his arrival in this country. It only remains to state, that by the time I succeeded in arresting his fever and diarrhœa, he was extremely emaciated, and his muscular system very much enfeebled.

It was now the early part of July, and I determined, after a careful consideration of the case, to attempt the reduction; at the same time, I was fully aware that, in order to compel the head of the bone to abandon its new position, to break up the adhesions which held it fast, and, finally, to replace it in its original situation, it was necessary to employ an amount of force, which seemed hardly justifiable in a constitution so debilitated. I appreciated the danger, and also the necessity for action; for the constant pain, augmented by the least movement, would have undoubtedly brought on a marasmus, terminating sooner or later in death.

The permanent contraction of the surrounding muscles, almost universally present under similar circumstances, was wanting in this case: as before stated, they were all in a flaccid condition, except the great gluteal, which was painful to the touch. The limb was somewhat more emaciated than its fellow of the opposite side, owing doubtless to the obstruction to the circulation and enervation, produced by adhesions of the vessels and nerves in their unnatural situation.

I did not think it prudent to submit the patient to the action of chloroform or ether, owing to his feeble condition, but was obliged to operate without the aid of these valuable means. The following was the method pursued:

The body being held by two assistants by means of two bands, one of which passed beneath the perineum, and the other under the axillæ, traction was made upon the limb by two strong and intelligent assistants. The movement of the head of the bone, resulting from this manœuvre, was very limited, even when the force was much increased; and the excruciating pain, which the patient referred to the iliac region, compelled us for the moment to desist.

The following day, the patient having obtained a tolerable night's rest by means of a narcotic potion, I concluded to attempt the reduction by flexion, believing that I could thus better prevent any accident which the necessary force might produce; the operator, in adopting this method, having it in his power to follow the head of the bone by pressure upon it with the hand, aiding its movement in the proper direction, or correcting any deviation that may occur. The emaciated condition of the boy was eminently favorable for such a procedure.

The patient being placed upon his back, and the trunk of the body made steady by assistants, with the left hand I grasped the upper part of the leg, placed the right hand upon the head of the bone in the iliac fossa, and then proceeded to flex the leg upon the thigh, and the thigh upon the pelvis. By this movement the great gluteal muscle was relaxed, and the head of the bone advanced, while with the right hand I directed the latter toward the cotyloid cavity. As soon as I judged the head to be immediately above the centre of the socket, I extended the leg, the thigh remaining flexed at a right angle; and then using the limb as a lever, I rotated it from within outward, and at the same time extended it by making a movement of circumduction in a similar direction. When by these procedures the limb was brought near to its opposite fellow, a snap, audible to the assistants, and of a deeper character than is ordinarily observed in the reduction of recent dislocations, indicated the return of the head of the bone to its natural position; a fact which was farther substantiated by the establishment of the original length and form of the member and the subsidence of the pain.

The after-treatment consisted in placing a pad between the knees, and another between the internal malleoli, and confining the limbs together by two bands, one above the knees, and the other around the lower part of the legs. But in spite of these precautions to prevent redisplacement, the next morning I found that the dislocation had been reproduced. It was again reduced, but for three successive days there was a redisplacement. After this, however, the head of the bone kept its place ; passive motion was daily employed, and all suffering ceased. After twenty days of rest, and a liberal use of the lactate of iron, the patient was allowed to get up; and, being provided with a pair of crutches, upon which he exercised himself daily, improved very rapidly. The muscles gradually recovered their bulk and vigor ; and at the end of forty-eight days he was enabled to walk without crutches, although with some fear of falling. About the middle of August, he was put to work in a cigar manufactory, and has continued well ever since.

*Remarks.*—Sir Astley Cooper remarks, that a surgeon should carefully consider before deciding to attempt a reduction of an old dislocation, especially in a young man. As a general rule, the following conditions are recognized as favorable to the operation. First, that the head of the bone is movable, as may be ascertained by making direct pressure upon it, or by moving the limb in various directions; secondly, that the situation of the head is such as to admit of subcutaneous incisions of the adhesions, if this should become necessary; thirdly, that the acetabulum is proved to be empty; fourthly, that the limb is shorter than its fellow; and, lastly, that the patient's general health is good. In the absence of any of these conditions, surgical authorities advise us against any violent attempt at reduction. In the case above reported, it was impossible to prove, whether or not, the cavity of the acetabulum was filled up, although I made the most careful examination; and as to the mobility of the head of the bone, the intolerable pain which accompanied the manipulations, enabled me to obtain this only to a very limited degree; moreover, the patient's constitution was in a very debilitated condition. Notwithstanding, however, these adverse circumstances,—circumstances which, we are advised to consider as forbidding any attempt at reduction,—I felt compelled to act, resting my judgment upon the fact, that the principal cause of the patient's rapidly failing health was the displacement of the bone. The result of the case proved the correctness of my opinion.

In reference to such cases, I have always adopted, as a diagnostic rule, that, so long as acute pain continues to be felt during movements of the limb, the great probability is, that the work of new formation is not complete, and, therefore, the cavity of the acetabulum not entirely obliterated. This circumstance was present in the foregoing case, and, as I considered the suffering as the cause of the patient's debilitated condition, it was my duty to remove the cause if possible. Besides, it was possible, indeed likely, that the enfeebled state of the system had delayed the formation of adhesions and a new cavity, and the contraction and transformation of the surrounding muscles. In proof of the latter, I need only refer again to the statement already made, that all the surrounding muscles, except the great gluteal, and, possibly, the triceps, were flaccid, and devoid of contractility; while, on the contrary, in the iliac region there was acute pain, with increased heat, discoloration, and tension of the skin, indicating the presence of inflammation, which sufficiently accounted for the fever sometimes observed. And it is reasonable to suppose, that, if the patient had not observed perfect rest, the increased pain would have given rise to more frequent exhibitions of fever. Thus, having discovered that the only point at which inflammation existed, corresponded to the situation of the head of the femur, it was natural to suppose, that the clo e contraction of the great gluteal muscle upon this foreign body was due to an effort of nature to get rid of the latter. The indication, therefore, seemed to be to aid, rather than to supplant the natural powers; but the contraction of the muscle upon the neck of the bone had produced an opposite effect, and it was necessary, therefore, to relax it, in order to remove the head from its abnormal position. For this purpose, I resorted to flexion of the thigh, seeing that traction upon the limb only increased the difficulty.

It is generally believed that no noise accompanies the reduction of an old dislocation, but in the foregoing case the sound was deeper than usual, and loud enough to be heard by all the assistants.

As soon as the great gluteal muscle was relaxed, the pain was much diminished; and when the bone entered the socket, the patient exclaimed that he was cured, and expressed a desire to get up immediately. But notwithstanding the relief thus suddenly obtained, the cure was by no means complete, for the muscles and ligaments, torn by the dislocation, had not the power to retain the head of the bone in its place, and hence the necessity for subsequent quietude of the limb. But with an impatient boy, tired of a six months' confinement to bed, this was no easy matter; and after several redisplacements and reductions, I determined to let him suffer a while, in order to impress upon his mind more forcibly the necessity of keeping quiet. The hope of accomplishing a perfect cure was, however, much increased by the greater ease with which each successive reduction was performed. Indeed, the last time that I had occasion to perform the operation, the reduction was accomplished by simply pushing the head of the bone back into the socket.

In conclusion, I may be permitted to state, that the period within which an old dislocation may be reduced cannot be fixed *a priori;* for some cases, which cannot be remedied by one method, may perhaps be by another. I would say that, so long as there is decided pain in the situation of the displaced bone, it is justifiable to attempt reduction; for the existence of pain indicates that the process of filling up the natural cavity, and the formation of a new articular surface are not complete. The objection may be urged that the natural cavity is no longer surrounded with ligaments capable of retaining the bone in its original position, but the same may be said of the new; and nature will certainly restore the necessary attachments sooner in a natural, than create them anew in an unnatural situation.

